# Quality of Life (QoL) among Health Care Workers with Diabetes Mellitus: A Literature Review

**DOI:** 10.3390/clinpract11040096

**Published:** 2021-10-30

**Authors:** Warda Alamri, Aisha Alhofaian, Nahed Mersal

**Affiliations:** Department of Medical and Surgical Nursing, Faculty of Nursing, King Abdul Aziz University, P.O. Box. 80209, Jeddah 21589, Saudi Arabia; aalhofaian@kau.edu.sa (A.A.); namali@kau.edu.sa (N.M.)

**Keywords:** health care workers, diabetes and work, diabetes mellitus, diabetic health care worker, diabetic employee, quality of life

## Abstract

Objective: This scoping literature review explores the impact of diabetes mellitus (DM) on the health-related quality of life (HRQoL) of health care workers (HCWs). HCWs play a vital role in the global health care system, with the COVID-19 pandemic demonstrating their effectiveness and worth beyond any doubt. However, HCWs are among the most vulnerable members of the health care system because they are most susceptible to stress, exhaustion, and occupational health risks. Method: The review was conducted in 2021 and included articles published in English in the past five years that explore diabetic HCWs’ QoL and studies intended to assess the relationship between work stress and DM. In total, 27 relevant articles were found that satisfied the inclusion criteria and were critically and thematically analyzed. Results: Most DM studies have focused on the clinical management of patients, but researchers have paid little attention to the high-risk group of HCWs with diabetes. In addition to fulfilling their job mandate, HCWs are burdened with various sociological stressors that affect their QoL. Conclusion: This literature review suggests DM has a significant impact on QoL in the work–life context. However, there is limited evidence to demonstrate the impact of DM on the QoL of HCWs. Thus, further research is needed in this area to improve the provision of integrated care.

## 1. Introduction

Health care workers (HCWs) constitute a vital component of the global health care system; the COVID-19 pandemic has proved their worth beyond a doubt. HCWs are also the most vulnerable components of the health care system because they are prone to stress, fatigue, and occupational health hazards. This vulnerability often makes them susceptible to various diseases and metabolic disorders such as diabetes mellitus (DM). The preexisting health complications of some HCWs further aggravate their vulnerability to such diseases. The prevalence of DM among HCWs deserves urgent attention in view of the serious health risks DM carries. These health risks can lead to a reduction in quality of life (QoL) and associated psychosocial complications in health care settings [1].

DM is defined as a group of metabolic disorders characterized by high blood glucose levels, defects in insulin secretion or action, or both. Thus, understanding DM and its complications plays a fundamental role in managing the disease and its spread. Furthermore, patients with proper knowledge of DM and DM complications follow a suitable treatment and health care plan [2].

DM has become a global problem. More than 180 million people have been diagnosed with the disease, and this number is expected to double by 2030. According to the World Health Organization (WHO), DM diagnoses are increasing steadily, and DM has become the seventh leading cause of death [2].

DM is considered a burden on people and countries because it results in renal failure, cardiac problems, vision loss, and limb amputation. It is estimated that approximately 7 million people have DM, and around 3 million are diagnosed with pre-DM [3]. In terms of DM rate, Saudi Arabia is considered second in the Middle East and seventh in the world.

### 1.1. QoL

There are several approaches to defining QoL. Some approaches depend on human requirements, expectations, subjective well-being, and phenomenological perspectives. In relevant literature, approaches based on objective lists, hedonism, preference satisfaction, life satisfaction, and flourishing are differentiated. Some of the ways in which QoL is defined are “a conscious cognitive judgment of satisfaction with one’s life” and “the way an individual perceives their position in life with respect to the culture and value systems in which they live and with respect to their expectations, goals, concerns and standards” [4].

Generic or disease-specific instruments may be used to measure health-related QoL. Generic instruments are widely applicable in different types and intensities of the disease as part of various health interventions and within demographic and cultural subgroups. The purpose of these instruments is to summarize the different health-related QoL (HRQoL) concepts that apply to various impairments, patients, diseases, and populations [5].

Disease-specific measures are those developed to examine particular diagnostic groups or patient populations. These methods are especially valuable when they concentrate on clinically significant changes. Disease-specific HRQoL utility evaluations may be used to perform cost-utility analysis (CUA) so that patients suffering from the same disease can be allocated resources [5].

The following domains are part of two widely used HRQoL measures: role constraints, physical functioning, social functioning, mental health, pain, vitality and mobility, self-care, usual activities, anxiety or depression, and pain or discomfort. These domains help with evaluating self-perceived health status [6].

The methods used to determine HRQoL typically include questions that may be divided into groups (domains or components) developed to examine particular issues that place constraints on health and well-being. Some of the most extensively used instruments for evaluating HRQoL are the World Health Organization Quality of Life Assessment (WHOQOL), the 12-item Short-Form Health Survey (SF-12), and the 36-item Medical Outcomes Study Short Form (SF-36) [7].

In this regard, QoL assessment instruments have played an important part in raising a question regarding the QoL of people suffering from chronic diseases, especially adults. A number of researchers have examined the relationship between chronic diseases and work-related QoL [7]. They found individuals suffering from chronic diseases reported relatively low QoL scores in the physical and/or mental health domains.

### 1.2. DM and QoL

DM is a significant issue in contemporary times. Meanwhile, QoL is a key therapeutic goal in managing the issues that have emerged from the pandemic. Much confusion still exists concerning the QoL context, DM-specific QoL, and HRQoL. Psychometric tools have recently been designed to evaluate QoL, DM-specific QoL, and HRQoL because DM affects the major QoL components. However, there are differences with respect to environment, ethnicity, culture, socioeconomic status, gender, lifestyle habits, profession, and diet [8].

Various factors (e.g., gender, age, physical activity, marital status, duration of DM, and presence of comorbidities) can affect diabetic patients’ QoL. Therefore, it is imperative to focus on the QoL assessment of diabetic patients and how various treatment approaches affect patients’ QoL [9].

Another study found relatively moderate HRQoL among adult patients suffering from type 2 DM (T2DM) in all domains. In this study, we found the physical domain is the most affected domain. There is a relationship between decreased HRQoL in all domains and overall HRQoL and disease duration, fasting blood sugar, and age. In addition, there is an inverse relationship between (a) overall HRQoL and (b) age, the prevalence of documented comorbidity, body mass index (BMI), comorbidities, blood glucose level, and disease duration [10].

Considering the relevance of DM and QoL in the management of long-term conditions, we aim to assess the QoL of HCWs with DM and the general effect of DM on working life in this review.

## 2. Materials and Methods

### 2.1. Search Strategies

**Figure 1:**
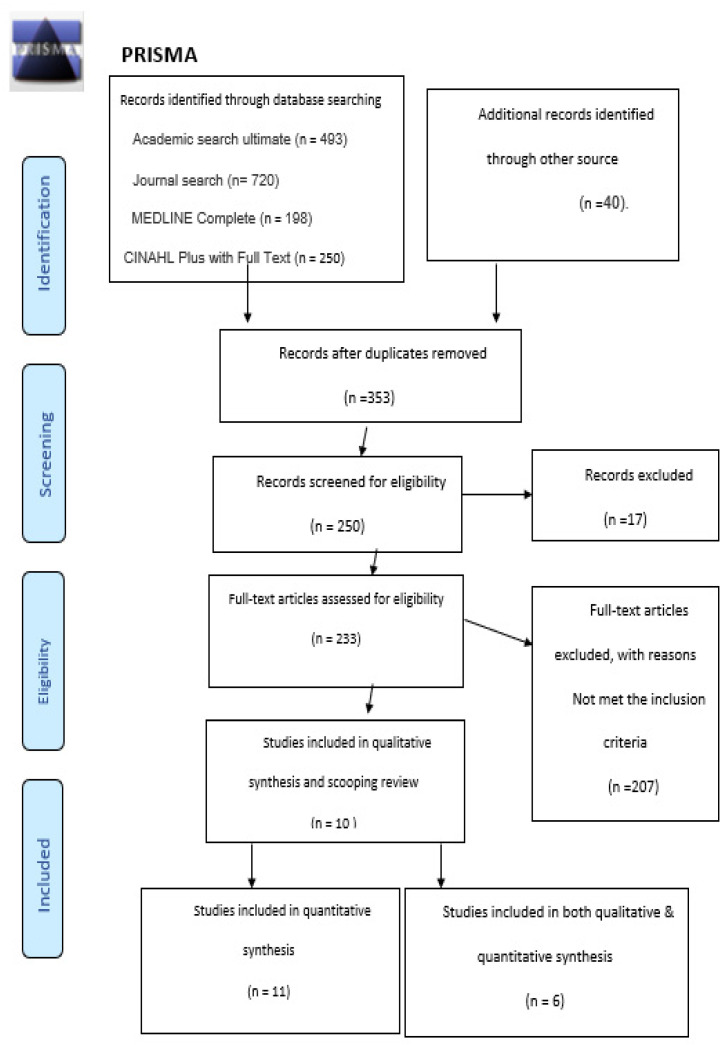
PRISMA Flow Diagram of Included Studies, adapted from Ref. [12].

In the present review, we began by identifying the appropriate inclusion criteria guided by the population, concept, and context (PCC) framework [11]. These criteria would produce relevant documents to meet the study’s objectives (Table 1). First, we identified the PCC to guide the database search process. Then, to identify articles relevant to the literature review, we conducted vigorous research for studies related to the QoL of HCWs, DM prevalence among HCWs, studies intended to assess the relationship between work stress and DM prevalence, and various factors that aggravate or alleviate the disease’s symptoms. The search terms included diabetes prevalence in HCWs, QoL among health care workers, diabetes and work, diabetes mellitus, diabetic health care worker, and diabetic employee quality of life. The initial search yielded 1701 studies, out of which we excluded studies that did not focus on DM and work. We included four studies as relevant to HCWs and included 20 studies as more pertinent to the general effect of DM on work and QoL. Out of these, we designated 24 studies for review as specific to DM-related issues among HCWs and employment, as outlined in Figure 1.

Finally, we created a thematic framework to guide and sort the existing literature and to collate, summarize, and report the results.

#### 2.1.1. Search Engines

We obtained the articles used in this review from the CINAHL, PubMed, Academic Search Ultimate, and Google Scholar databases using the keywords (search terms) listed in Table 2. The search terms included diabetes prevalence in HCWs, HCWs, DM and work, DM, diabetic HCW, and diabetic employee QoL.

#### 2.1.2. Inclusion and Exclusion Criteria

##### Inclusion Criteria

The inclusion criteria for the review were literature published between 2015 and 2021; studies, reports, and published articles that focused on QoL of HCWs and DM prevalence among HCWs; studies intended to assess the relationship between work stress and DM and various factors that aggravate or alleviate the disease’s symptoms; articles published in the English language; peer-reviewed review articles, including systematic reviews, meta-analyses, and scoping reviews; rapid reviews; and grey literature sources, such as documents from the government and nongovernmental organizations and academic dissertations.

##### Exclusion Criteria

The exclusion criteria were articles not published in the English language and articles published before January 2015.

#### 2.1.3. Study Selection Process

We restricted the research criteria to studies conducted from 2015 to 2021. During the initial search, we discovered 1701 articles. We preferred this time restriction because of the need to identify the most recent discussions on the subject. According to the aforementioned inclusion criteria, the studies included keywords and were examined first based on title, then abstract, and then overall content. The flow diagram (Figure 1) is a diagrammatic description of the search process and the criteria for the articles chosen. It summarizes the literature search and article selection process. Finally, we selected 27 of the articles to include in this review.

#### 2.1.4. Quality Assessment of Included Studies

Two expert reviewers used the quality scoring system tool formulated by [13], cited in [14], to examine the quality of the studies considered. We included nine components in the evaluation to analyze the research: the title and abstract, introduction and aim, methodology and data sampling, data analysis, bias and ethics, findings, transferability, implications, and utility. Based on the research quality, we classified the scoring as “good”, “fair”, “poor”, or “very poor”, with points ranging from 4 for good and 1 for very poor. This generated a minimum score of 9 points and a maximum of 36 points for every study. The definitions given below were used to generate the overall quality grades: high quality (A) with 30–36 points, medium quality (B) with 24–29 points, and low quality (C) with 9–24 points [14]. We conducted the evaluations as depicted in Table A2. In the final quality assessment, we obtained high-quality results in 25 of the studies, medium-quality results in one study, and poor-quality results in one study.

## 3. Results

We will present an explanation of the selected articles in the following subheadings, which will explain each study’s characteristics more specifically. All included studies were reviewed separately based on their title, design, sample size, setting, and main finding, as shown in the review matrix in Table A1. The matrix helped us find suitable themes and discuss the selected studies based on the analysis in the literature review presentation section.

The findings from the 27 articles selected for review were reported as main thematic analysis themes and factors that affect diabetic workers’ QoL. Because there were few studies about QoL among diabetic HCWs, only two studies deliberately focused on HCWs’ QoL, whereas four studies discussed the prevelance of DM among HCWs. Nena et al., 2018 [15] and Muthuri et al., 2021 [16] conducted cross-sectional studies on HCWs’ QoL. Coetzee et al., 2019 [17] conducted a retrospective analysis to assess the contribution of traditional and modifiable risk factors to the overall risk and prevalence of T2DM among HCWs in the public sector [18]; the cross-sectional study assessed only the prevalence of DM, hypertension, and obesity among doctors and nurses in a tertiary care medical college hospital in Tamil Nadu, India. Huang et al., 2016 [19] conducted a retrospective and longitudinal study on whether the incidence risk of T2DM between female nurses and female non-nurses differed. Hansen et al., 2016 [20] conducted a cohort study to examine the relationship between shift work and the incidence of DM among Danish nurses.

The remaining 21 studies reviewed the QoL among diabetic patients and the general impact of DM on working life (Table A1). Out of 21 studies, 13 were cross-sectional studies carried out by Manodpitipong et al., 2017 [21], Nakao et al., 2021 [22], Olesen et al., 2020 [23], Seuring et al., 2015 [24], Hansen et al., 2018 [6], Pasmooij et al., 2016 [25], Hakkarainen et al., 2016 [26], Loerbroks et al., 2018 [27], Sonoda et al., 2020 [28], Nielsen et al., 2016 [29], and Tonetto et al., 2019 [30]. A comparative cross-sectional study was carried out by Binesh et al., 2021 [31], whereas Abu et al., 2016 [32] performed a case-control study. A scoping literature review was carried out by Galarraga and Llahana, 2018 [33] in England, whereas Gerbo et al., 2019 [34], Smith et al., 2018 [35], and Imbroll and Cassar, 2021 [36] employed a qualitative exploratory approach. Retrospective cohort studies were conducted by Nexø et al., 2020 [37], Ervasti et al., 2015 [38], and Ervasti et al., 2016 [39], whereas McCarthy et al., 2021 [40] used a convergent mixed-method approach to conduct a study in the United States.

Regarding the countries the selected studies focused on, six studies were conducted in Denmark, three in the United Kingdom one in Germany two in the United States, two in Japan, one in Iran, one in Malta one in Egypt, one in Finland, one in Thailand, one in Mexico, one in Taiwan, one in India, two in South Africa, and one in Sweden; the last were conducted in three European regions: northern (Sweden, Denmark), central and western (the Netherlands, Belgium, Germany, Austria, Switzerland, and France), and southern Europe (Italy, Spain, and Greece). One study was conducted in Greece, and one was carried out in Brazil.

Nine articles used a quantitative method, and 10 used a qualitative method. However, two scoping reviews, one systematic review, and six studies used both a qualitative and a quantitative method.

## 4. Discussion

Following the thematic analysis, we found four recurring themes that helped us identify the overall effect of DM on HCWs’ QoL and that of diabetic employees in general.

### 4.1. QoL among HCWs and Diabetic Patients

To overcome the challenges in the health care system, improve the quality of care, and increase patient satisfaction with the care received, it is important to know how satisfied HCWs view their QoL and job and what characteristics influence their QoL. This review revealed the overall perception of QoL among HCWs.

As Tonetto et al., 2019 [30] noted, some personal, job-related, and work environment predictors and characteristics are significant predictors of QoL among HCWs. These predictors could be related to HCWs’ professional roles, which significantly affect QoL. In their study, nurses reported lower HRQoL scores compared to doctors and occupational safety and health technologists.

Shift work also has a significant impact on QoL. In a study performed in Greece that included 312 employees (87.9% female), 194 working irregular shifts and 118 on morning shifts, 58.2% were somewhat or totally dissatisfied with their sleep quality. DM was the most common medical condition that shift workers reported (*p* = 0.008). A comparison of the two groups revealed significant impairment in QoL; in addition, disease duration and frequency, along with employees’ age and family status, can have adverse effects on sleep quality [16].

Understanding how DM can compromise a person’s QoL enables identification of care needs and thus contributes to improving QoL and control of the disease. The results of the present study suggest the QoL of a person with DM may worsen as care for the disease becomes more complex—QoL tends to worsen as the disease worsens. The results suggest QoL is related to sociodemographic and clinical variables that should be considered in care [15].

### 4.2. Overall Risk Factor and Prevalence of DM among HCWs

The prevalence of DM among doctors and nurses was evaluated by Huang et al., 2016 [19]. The findings showed a 25.4% prevalence of DM among doctors and 5.6% among nurses. The findings of Hansen et al., 2016 [20] demonstrated that compared to non-nurses, nurses are at lower risk of developing DM, which may be because of their medical knowledge and educational training. Moreover, nurses undertake health promotion and educational responsibilities.

The same is true with the cohort study conducted in Denmark. Out of 19,873 nurses who worked and were DM-free at recruitment, 837 (4.4%) developed DM during 15 years of follow-up, with a statistically significant number working the evening or night shifts. This demonstrated that night shift nurses were more at risk of developing DM than those working the day shift [21].

Conversely, a study of 260 HCWs with DM in South Africa found 62 nurses (18%) had hyperglycemia. Therefore, to ensure the well-being of HCWs and society, there is a need to focus on healthy living, learning, and the health risks of obesity [18].

### 4.3. Overall Impact of DM on Work and Employee Productivity

The question of diabetic employees’ ability to be productive is essential in work productivity and job satisfaction evaluation. A major contrast was seen between a patient suffering from DM and hypertension in a study by Galarraga and Llahana, 2018 [33], where diabetic patients were found to be more affected with problems such as loss of function, efficiency, and work absenteeism. The job contentment level was low among diabetic patients compared to hypertensive patients because DM seems to lessen a person’s work capacity.

A study by Ervasti et al., 2015 [38], in which both men and women were studied individually, showed substantially more Hazard Ratios of sickness leave were seen in both men and women suffering from type 1 DM (T1DM) or T2DM than those without the disease. Although there was no significant difference between those with T1DM and T2DM, men with DM had significantly higher risks of disability pension, unemployment, and sick leave than women.

The benefits received by people suffering from cardiovascular disease (CVD) or DM are linked to early retirement, per long-term research. Another identified reason that leads to quitting work involves greater job requirements alongside fewer benefits. Therefore, it was recommended that optimizing psychosocial work-related factors could be beneficial for people with CVD or DM [26].

This aligns with the outcome of Kouwenhoven-Pasmooij et al., 2016 [25], who found DM indicates a considerably less productive workforce in men and women with DM in Mexico. For this reason, DM has been associated with a fall in the productive workforce and the economy, which suggests DM represents a large burden for people in Mexico. However, a research study performed on six employees and two managers suffering from DM showed their condition had no major adverse effects on their ability to function daily, and they could perform their routine tasks easily; in fact, DM is highly unlikely to impose substantial effects on an individual’s ability to pursue a particular career, and the employer is usually unaware of the employee’s condition [37].

Hansen et al., 2016 [20] discovered the same. They assessed the working capability of adult diabetic patients and found the results satisfying. Moreover, half the patients reported suffering no adverse effect on their DM self-management. Therefore, it is recommended that social welfare and work–life harmony (e.g., involving diabetic workers in learning programs) can improve working capacities.

Work disability was substantially higher among people with DM (overall mean = 95 days per year over 7 years, SD = 143) than among those without DM. In addition, the risk of work disability was slightly higher after DM diagnosis than before and compared with the risk of those without DM [39].

### 4.4. Sociodemographic Analysis and Affected QoL Domains

#### 4.4.1. Sociodemographic Analysis

Age and obesity are major sociodemographic factors that contribute to DM. Huang et al., 2016 [19] found the prevalence of obesity was 15.1% among doctors and 3.2% among nurses, which was found to be statistically significant. In addition, the crucial factors of the risk of DM occurrence in nurses are age and the Charlson Comorbidity Index (CCI) scale, which predicts the 10-year survival rate for patients with multiple comorbidities, as discovered by Hansen et al., 2016 [20].

Shift work can be one of the main reasons for increased incidence of DM, according to Manodpitipong et al., 2017 [21]. They found no evidence of an interaction between night-shift work and BMI, which suggests that in terms of DM risk from shift work, weight gain was more common among nurses working night shifts than day shifts. One of the main reasons for weight gain attributed to night-shift work is that excessive secretion of cortisol and interleukins, together with increased insulin concentrations, can lead to abdominal fat buildup, lipid disorders, and insulin resistance.

Furthermore, various other factors can lead to obesity; these include bad diet and inadequate sleep from working night shifts, as identified by Nakao et al., 2021 [22]. They found that patients with T2DM have excessive BMIs. Per the HbA1c estimate, they had poor glycemic control compared with participants working day shifts or unemployed participants.

In addition, work schedules, including rotating shifts, can affect the timing of medications and nutritional intake, thus making it more challenging for individuals to effectively manage the condition. Moreover, time away from work may be necessary for medical appointments or illness [35].

In one study, half of the participants, who were 55 years or older (47%), were ranked in the highest-risk group, and 37% of the participants in the high-risk group were older than 55 years [16]. Increasing age adversely affects lifestyle choices, work status, and health, especially among women [31].

In their study, Ervasti et al., 2015 [38] found workers with DM in Sweden were more likely to have other risk factors of work disability and leave absence, including older age, a lower education level, and comorbid conditions, than those without DM. DM risks for employees increase at age 44 and are greater for both men and women [23].

#### 4.4.2. Affected QoL Domains

According to [41], there are eight QoL domains: physical functioning (PF), physical role limitations (PL), bodily pain (BP) energy/vitality (VT), general health perceptions (GH), social functioning (SF), emotional role limitations (RE), and mental health (MH).

In this review, we found the most common QoL domains affected were predominantly related to physical health, emotional well-being (particularly anxiety and occupational stress), and social functioning. Compared to the general population, adults with T1DM experienced lower health-related QoL [31].

Diabetic workers face multiple challenges every workday—diet, exercise, and patient education. Furthermore, the treatment of individuals with T1DM, with its total dependence on exogenous insulin, includes injections of basal and prandial insulin or continuous subcutaneous insulin infusion. In addition, work schedules, especially rotating shifts, can affect the timing of medications and nutritional intake, thus making it more challenging for individuals to effectively manage the condition. Time away from work may be necessary for medical appointments or illness [35]. Of 140 employees with DM aged over 40 years, 12 (8.6%) were unable to attend their appointments and were considered the dropout group of outpatients [29].

Many factors lead to people quitting their jobs and taking disability allowances, including excessive and physical workload requirements. However, this is more common in employees with DM or CVD [24], which highlights the importance of assistance and training from management at work. For example, using adjustable working arrangements and timing schedules to align with employees’ well-being are essential to improving work efficiency [37].

According to [6], young people with T1DM experience a great deal of presure and find it difficult to manage their condition because of their higher work demand. Thus, this may interfere with their DM management. In the same study, a participant confessed that because of his workload, he does not focus on his DM management and skips his breaks—actions that can lead to a further increase in stress levels.

According to [27], when work requirements go beyond an individual’s resources for managing their work, they may experience work-related stress—a major cause of burnout—depression, and anxiety. Furthermore, anxiety, depression, burnout, and low working capacity have been linked to self-admitted fatigue. Anxiety is an important comorbidity factor to be considered in people with DM, although the systematic review indicated DM may not be associated with an increased risk of incident anxiety [36]. However, there is still evidence that anxiety is associated with poorer outcomes in people with DM, such as poorer glycemic control, worsened functioning, and increased DM complications.

There is a significant relationship between changing shift patterns and nighttime shifts and having T2DM. The relationship is often characterized by poor glycemic control that is rarely found in people who do not work in shifts [20]. Changing shift patterns affect eating and sleeping patterns, thus altering blood glucose levels, disturbing circadian rhythms, and causing a reduction in insulin sensitivity that further increases problems in daily routines [23].

Psychosocial factors trigger stress-associated hormonal pathways, which then disturb DM management and glucose metabolism and alter lifestyle. The psychosocial factors that affect the clinical and social results of DM include psychological strain, job stress, and social support [40].

One of the challenges that employees with DM face every workday is the tension between the worker’s logic and the patient’s logic. According to Hansen et al., 2018 [6], a participant said she “has accepted that she avoided taking insulin at her work due to the tasks’ uncertainty at her work[;] she considered it a safer option to have high levels of glucose in her blood rather than going through hypoglycemia at her work place where children are involved”.

T1DM patients develop work-related stress like any other worker, although theirs is usually compounded by the stress of self-managing the disease [27]. Agreeing with this observation, McCarthy et al., 2021 [40] found people with DM often go through work with a disability, which has been linked to psychological distress.

Distress has been linked to biopsychological factors associated with occupational balance in people with DM. An increasing level of distress is associated with lower occupational balance and poorer glycemic control among diabetic employees [32]. Seuring et al., 2015 [24] found that compared with other working populations, an overall poor psychological working atmosphere has been noted in employees with T2DM.

Moreover, many studies mentioned the effectiveness of work-related support and social support in creating better work–life balance for diabetic workers. Additionally, keeping shift patterns and hours in mind when offering time management support to diabetic workers is essential; this could enhance workers’ QoL and work capabilities and better their work–life balance. In terms of work-related factors, one study [29] found greater supervisor support (emotional and physical support) was associated with a lower risk for dropping out of outpatient visits for DM treatment among Japanese male employees with DM.

## 5. Gap and Limitations of the Included Literature

By reviewing the literature, we found some limitations with the search strategy when applying keywords (the search terms) as a result of limited studies, systematic reviews, and meta-analyses conducted on this topic globally and locally in Saudi Arabia. To the best of our knowledge, this review is one of few exhaustive reviews. However, we included studies related only to HCWs. The second limitation is the search scope; we focused on recent studies. With these limitations, we cannot generalize findings and draw concrete conclusions for recommendations.

## 6. Summary

The main purpose of this scoping review was to identify an overview regarding the QoL of HCWs with DM. There have been limited studies, systematic reviews, and meta-analyses conducted on this topic globally, but we believe this review is exhaustive.

After thematic analysis, we found age and obesity were the major sociodemographic factors contributing to DM. Furthermore, we found emotional wellness, physical well-being, and social performance (particularly anxiety and occupational stress) are the primarily influenced QoL domains. Although the overall impact of DM on work productivity was significantly high, the job satisfaction score was relatively low, and work absenteeism was incredibly high. In addition, work productivity loss and impairment were more common among diabetic employees.

However, intervening with social support, providing work–life balance, and involving diabetic workers in educational programs are strategic efforts that occupational health staff can use to avoid the aforementioned problems. Additionally, keeping shift patterns and hours in mind when offering time management support to diabetic employees is essential because doing so can enhance their QoL and work capabilities.

## Figures and Tables

**Table 1:** PCC and Inclusion and Exclusion Criteria.

PCC
Population: Diabetic HCWs and diabetic employees
Concept: QoL of HCWs, DM prevalence among HCWs, assessment of the relationship between work stress and DM prevalence, and various factors that aggravate or alleviate the disease’s symptoms
Context: QoL among diabetic HCWs

**Table 2:** Key Search Terms.

Key Search Terms
(Diabetes Mellitus) OR (Diabetic Quality of Life) AND (Health Care Worker) OR (Diabetes Mellitus) AND (Employee) OR (Diabetes and Work).

## Data Availability

Not applicable.

## Appendix A

**Table A1:** Literature Review Matrix.

Author(s)/ Year of Publication	Research Setting(s)	Tool(s) Used	Study Group(s)	Methodology	Result(s)
(1) Ankia Coetzee, Amanda Beukes, Reinhardt Dreyer, Salaamah Solomon, Lourentia van Wyk, Roshni Mistry, Magda Conradie, and Mari van de Vyver, published in 2019 [17]	Tygerberg Hospital, Cape Town, South Africa	Test2Prevent and risk stratification	All health care workers (HCWs) at Tygerberg Hospital	A retrospective analysis was performed on data obtained from 260 participants.	Education, lifestyle, and overweight interventions are of paramount importance to ensure the metabolic health of HCWs and their communities. Policies and guidelines focused on limiting unhealthy/obesogenic work environments are urgently needed. The prevalence of known hyperglycemia in this cohort is concerning (11%, *n* = 62). An additional 29 HCWs were identified as at high risk of developing type 2 diabetes mellitus (T2DM) within 10 years. Consumption of sugar-sweetened beverages and minimal physical activity were identified as modifiable intervention targets.
(2) Shailendra Kumar B. Hegde, S. Sathiyanarayanan, Sathiya Narayanan, Sanjana Venkateshwaran, Akshaya Sasankh and P. Ganeshkumar, Balaji Ramraj, published in 2015 [18]	Tamil Nadu, India	Case record form developed	Doctors and nurses aged 18–65 years working at a tertiary care medical college	This was a cross-sectional study; the study period was June–August 2013.	The prevalence of diabetes mellitus (DM) was found to be significantly higher among doctors (25.4%) than nurses (5.6%). In addition, the prevalence of hypertension was found to be significantly higher among doctors (29.4%) than nurses (13.7%). Other factors such as age and obesity were covered.
(3) Hsiu-Ling Huang, Cheng-Chin Pan, Shun-Mu Wang, Pei-Tseng Kung, Wen-Yu Chou, and Wen-Chen Tsai, published in 2016 [19]	National Health Insurance Research Database, Taiwan	Modified Charlson Comorbidity Index (CCI)	Nurses	A retrospective and longitudinal method was adopted in this study, and data were sourced. To comply with privacy measures, personal information was removed from the collected data. Study data were obtained from the Longitudinal Health Insurance Research Database, and nurses were sampled from the Registry for Medical Personnel. Nurses and non-nurses with similar traits and health conditions were selected via one-to-one propensity score matching. A total of 111,670 subjects were selected (55,835 nurses and 55,835 non-nurses).	The authors found found age and CCI serve as critical factors that influence DM development risks among nurses. The low degree of DM development among the nurses may be attributable to the fact that nurses possess substantial knowledge on health care and on healthy behaviors. The results of this study can be used as a reference to assess occupational risks facing nursing staff, to prevent DM development, and to promote health education.
(4) Ulla Møller Hansen, Bryan Cleal, Ingrid Willaing, and Tine Tjørnhøj-Thomsen, published in 2018 [6]	Diabetes Clinic of the Danish Denmark	In-depth interview as the method of inquiry	Employees with type 1 diabetes mellitus (T1DM)	A total of 40 interviews were conducted using the Atkinson analysis. The first phase was within-case analysis; the second phase was theory-based analysis. During the third phase, we refined the analysis.	Managing T1DM in work–life may be articulated as a matter of containment: the assemblage of practices and mental and emotional work aimed at keeping DM at the level of a side involvement. Workers with T1DM face tension between the worker’s and patient’s competing logics, which temporality heavily influences.
(5) Till Seuring, Yevgeniy Goryakin, and Marc Suhrcke, published in 2015 [24]	Mexican Family Life Center, Mexico	Mexican Family Life Survey (MxFLS)	Diabetic employees in Mexico	A nationally representative household survey was conducted in 2002 and 2005. We used data from the second wave in 2005, which included almost 40,000 individuals. Descriptive statistics were used.	We found that DM significantly decreases employment probabilities for men by about 10 percentage points (*p* < 0.01) and somewhat less so for women (4.5 percentage points, *p* < 0.1) without any indication of DM being endogenous. Further analysis shows DM mainly affects the employment probabilities of men and women above the age of 44 and has stronger effects on the poor than on the rich, particularly for men. We also found some indication for more adverse effects of DM on those in the large informal labor market than those in formal employment. Our results highlight—for the first time—the detrimental employment impact of DM in a developing country.
(6) T.A. Kouwenhoven-Pasmooij, Alex Burdorf, J.W. Roos-Hesselink, M.G.M. Hunink and. S.J.W. Robrock, published in 2016 [25]	Three European regions: northern (Sweden and Denmark), central and western (the Netherlands, Belgium, Germany, Austria, Switzerland, and France), and southern Europe (Italy, Spain, and Greece)	Survey of Health and Retirement in Europe (SHARE)	Employees with cardiovascular disease (CVD) and/or DM	Longitudinal survey of 5182 employees	The results of this longitudinal study showed that having CVD or DM is associated with early exit from paid work through disability benefits or early retirement, and having low rewards or high job demands with low control further increased this probability. It showed that optimizing psychosocial work-related factors could be beneficial in people with CVD or DM.
(7) Sofia Llahana and Michele Miranda Galarraga, published in 2018 [33]	University College London, England	Scoping literature review	The career QoL of adults with T2DM	The review was conducted in February 2015 and included articles published in English in the past 10 years exploring career quality of life (QoL). Five articles satisfied the inclusion criteria and were critically and thematically analyzed.	Evidence suggests emotional well-being is the QoL domain that T2DM affects most, with depression and anxiety being reported as the most common issues. Additional domains were social functioning and economic burden. Sociodemographic and cultural differences were identified as variables that can influence a career’s QoL. This literature review suggests T2DM in adults has a significant impact on their career’s QoL; however, there is limited evidence to demonstrate how health care professionals can support careers, so further research is needed in this area to improve the provision of integrated care.
(8) Areesa Manodpitipong, Sunee Saetung, Hataikarn Nimitphong, Nantaporn Siwasaranond, Thanawat Wongphan, Chotima Sornsiriwong, Pranee Luckanajantachote, Prasitchai Mangjit, Prasit Keesukphan, Stephanie J. Crowley, Megan M. Hood, and Sirimon Reutrakul, published in 2017 [21]	Ramathibodi Hospital, Mahidol University, Bangkok, Thailand	Depressive symptoms were assessed using the Thai version of the Center for Epidemiologic Studies-Depression Scale. We used the modified Pittsburgh Sleep Quality Index (PSQI) score to assess sleep quality independently of sleep duration. Morningness–eveningness preference was assessed using the validated Thai version of the composite morningness score. Participants were interviewed regarding their dietary intake from the previous day (24-h dietary recall).	A total of 249 adult participants with T2DM who worked night shifts in six hospitals in Thailand were invited to participate.	We compared glycemic control in patients with T2DM who were performing night-shift work compared with those who were non-shift workers and those who were unemployed. Sleep duration, sleep quality, and dietary intake were examined as potential confounders. In addition, morningness–eveningness preference, previously reported to be related to worse glucose control in patients with T2DM, was considered.	Night-shift work is associated with poorer glycemic control in patients with T2DM. Thus, reducing the adverse metabolic effects of circadian misalignment may help improve glycemic control in this patient group. We found night-shift workers had higher body mass index (BMI) than unemployed participants
(9) Pirjo Hakkarainen, Leena Moilanen, Vilma Hänninen, Jarmo Heikkinen, and Kimmo Räsänen, published in 2016 [26]	The University of Eastern Finland and the Kuopio University Hospital, Finland	The questionnaire was constructed based on previously validated work–life scales and DM questionnaires.	Workers with T1DM	This was a cross-sectional study. The questionnaire was mailed to a random sample of 2500 Finns with T1DM aged 18–65 years and drawn from the Medication Reimbursement Register of the Social Insurance Institution of Finland; 767 respondents were included in the analysis.	Work-related DM distress was found to be common among workers with T1DM. Problems with physical work conditions, work ability, difficulty in accepting DM, job demands, and depressive symptoms proved to be associated with work-related DM distress.
(10) Mahmoud E. Abu Salema, Nagwa N. Hegazy, and Shaimaa G. Mohamed, published in 2016 [32]	The urban area was the primary health care unit of Shebin EL-Kom City, Shebin District, Menoufia Governorate. The rural area was the primary health care unit of Batanon Village, Shebin EL-Kom District, Menoufia Governorate, Egypt	Arabic-validated version of work productivity and impairment, the general health version (WPAI: GH), and job satisfaction questionnaire	Nurses and midwives	This was a case-control cross-sectional study. A total of 800 participants were recruited (400 patients and 400 controls). They were enrolled from urban and rural family health units as follows: 223 DM patients, 177 hypertensive patients, and 400 control group participants.	Work absenteeism, work productivity loss, and work impairment were more prominent with diabetic patients than with hypertensive patients. There was a statistically significant difference between the studied groups and job satisfaction; the job satisfaction score was lower for diabetic patients than for hypertensive patients. DM appears to reduce an individual’s ability to work more than hypertension does.
(11) Claire Farrugia Imbroll and Maria Cassar, published in 2021 [36]	University of Malta, Malta	Semi-structured, face-to-face, individual interviews were conducted. The sample consisted of six employees and two managers of an organization based in Malta.	Employees with DM	A qualitative exploratory approach using one-to-one interviews was adopted. Thematic analysis was used to analyze the data, which was carried out in keeping with Miles and Huberman’s model.	We explored the supports and challenges that adults with DM experience when fulfilling their employment commitments. We hope the findings will contribute to enhancing employees’ experience with DM at their workplace as well as inform employers and guide policy development.
(12) Jenniy Ervasti, Mika Kivimäki, RosemaryDray-Spira, John Head, Marcel Goldberg, Janna Pentti, Markus Jokela, Jonnas Vahtera, Marie Zins and Matti Virtanen, published in 2015 [39]	Finnish Institute of Occupational Health, UK	The study used standard questionnaires, and study-specific estimates were pooled using fixed-effects meta-analysis.	Employees with DM	The study involved a pooled analysis of individual-participant data from three occupational cohort studies (the Finnish Public Sector Study, the British Whitehall II Study, and the French GAZEL Study). A total of 1,088 women and 949 men with DM were followed up with to determine the duration and frequency of their work disability. The mean follow-up periods were 3.2 years in the GAZEL Study, 4.6 years in the Whitehall II Study, and 4.7 years in the Public Sector Study.	Psychological distress was associated with increased duration and frequency of work disability among employees with DM. Job strain was associated with increased absence frequency but not with absence duration.
(13) Kasper Olesen, Bryan Cleal and Ingrid Willaing, published in 2020 [23]	Steno Diabetes Center, Copenhagen, Denmark	Single self-reported items were used to obtain information about DM via a status survey.	Employees working with T2DM	This study was based on survey data from 2415 working Danes with T2DM (*n* = 586) and without T2DM (*n* = 1829) recruited from online panels. Single self-reported items were used to obtain information about DM status, exposure to discrimination, and other individual factors.	People with T2DM reported a relatively poor psychosocial working environment compared with the general working population, but the difference was removed by adjusting for overweight/obesity. This indicates T2DM alone is not a source of stigma and discrimination in the work context. Levels of perceived discrimination were notably lower than expected among people with DM as a whole, but many people still continue to be exposed to the destructive effects of discrimination in the work context.
(14) Mette Andersen Nexø, John Pedersen, Bryan Cleal and Jakob B. Bjorner, published in 2020 [37]	Danish National Patient Register, Denmark	Multi-state Cox proportional hazards analyses	Employees working with DM	This was a retrospective cohort study. Individuals with T1DM (*n* = 431) and T2DM (*n* = 4047) were identified in Danish national registers from 1994 to 2011 and compared with individuals without DM (*n* = 101,295). Multistate Cox proportional hazards analysis estimated hazard ratios (HR) with 95% confidence intervals (CI) for transitions among work, sickness absence, unemployment, and disability pension.	Both T1DM and T2DM affect labor market outcomes, but future studies also should consider comorbidity and social gradient.
(15) Maryam Binesh, Rokhsareh Aghili, and Afsoon Hassani Mehraban, published in 2021 [31]	Institute of Endocrinology and Metabolism, affiliated with Iran University of Medical Sciences, Iran	Diabetes Distress Scale and Life Balance Inventory	A total of 160 individuals (80 people with DM and 80 without DM)	A comparative cross-sectional study was conducted using simple nonprobability sampling. The Life Balance Inventory evaluated participants’ occupational balance. Blood samples were taken from those with DM and analyzed. Psychological distress was also evaluated in people with DM using the Diabetes Distress Scale.	Distress was the only biopsychological factor associated with occupational balance in people with DM. A higher level of distress is associated with lower occupational balance and poor glycemic control in this population.
(16) Tomomi Nakao, Chizuko Takeishi, Chiyo Tsutsumi, Yuichi Sato, Yuji Uchizono, and Yasuko Shimizu, published in 2020 [22]	Outpatient clinics at three general hospitals and three clinics in Japan	The Daily Time Management Scale for Use by Working People with Type 2 Diabetes	A total of 277 working people with T2DM	A descriptive cross-sectional study using a questionnaire survey was administered to 277 working people with T2DM. It included a daily time management scale and questions about age, gender, hemoglobin A1c levels, shift work, managerial position, and average working hours. Multiple regression analysis was used to assess the relationship between daily time management and each factor, adjusted for age, gender, and hemoglobin A1c.	When providing time management support to working people with T2DM, any assessment should consider the availability of shift work, whether employees are in a managerial position, and their work hours.
(17) Margaret McCarthy, Allison Vorderstrasse, Joeyee Yan, Angie Portillo, and Victoria Vaughan Dickson, published in 2021 [40]	Local academic medical center and Research Match, US	Work Ability Index and Karasek’s Job Content Questionnaire (JCQ)	A total of 101 employees with DM	This study used a convergent mixed-method design. We assessed the relationship between work-related factors and work ability using bivariate statistics and logistic regression. Work ability was measured using the Work Ability Index, and Karasek’s JCQ was employed to measure job demands. Qualitative interviews explored the relationship between DM and work.	Social support and work–life balance are associated with excellent work ability. Therefore, engaging workers with DM in workplace educational programs may take strategic efforts by occupational health staff.
(18) Helena B. Nielsen, Louise L. Ovesen, Laust H. Mortensen, Cathrine J. Lau, and Lene E. Joensen, published in 2016 [29]	Steno Diabetes Center, Capital Region in Denmark	Danish National Health Survey (“How Are You?”)	Employees with T1DM were compared to employees without DM.	A total of 2,415 adults (aged 18–98 years) with T1DM were compared to 48,511 adults (aged 18–103 years) from the general population. Data were obtained from two cross-sectional surveys conducted in 2010 and 2011 of adults living or treated in the Capital Region in Denmark. Differences between adults with T1DM and the general population were standardized for age and sex and analyzed using linear probability models and negative binomial regression.	Compared to the general population, adults with T1DM experienced lower health-related QoL (HRQoL), were more frequently unemployed, had more sick leave per year, and were slightly better educated. Differences in HRQoL and employment increased with age and were greater among women than men. No significant differences were found with regard to working hours.
(19) Adrian Loerbroks, Xuan Quynh Nguyen, Patricia Vu-Eickmann, Michael Krichbaum, Bernhard Kulzer, Andrea Icks, and Peter Angerer, published in 2018 [27]	Diabetes Center Mergentheim, Germany	In-depth interviews in face-to-face contact or by telephone	A total of 30 employed adults with DM	A total of 30 employed adults with DM living in Germany (*n* = 19 with T1DM, *n* = 11 with T2DM, 57% female, aged 24–64 years) were recruited. Using a topic guide, we carried out in-depth interviews in face-to-face contact or by telephone. Interviews were transcribed and content-analyzed using MaxQDA.	Various types of occupational psychosocial factors may determine DM self-management practices at the workplace. Quantitative studies are needed to confirm our observations. Subsequently, interventions could be developed and evaluated to improve opportunities to adequately engage DM self-management at work.
(20) Anne B. Hansen, Leslie Stayner, Johnni Hansen, and Zorana J Andersen, published in 2016 [20]	Danish Diabetes Register for incidence of DM, Denmark	Cox proportional hazards model	Nurses	We used the Danish nurse cohort, with 28,731 participating female nurses recruited in 1993 (19,898) or 1999 (8833), when self-reported baseline information on DM prevalence, lifestyle, and working time were collected. We followed them in the Danish Diabetes Register for incidence of DM until 2013. Nurses reported whether they worked night, evening, rotating, or day shifts. We analyzed the association between working time and DM incidence using the Cox proportional hazards model adjusted for DM risk factors, separately with and without adjustment for BMI, which might be an intermediate variable.	Danish nurses working night and evening shifts have an increased risk for DM, with the highest risk associated with current night-shift work.
(21) Robert M. Gerbo, Chuan Fang Jin, and Karen Clark, published in 2019 [34]	Department of Occupational and Environmental Sciences, USA	Review	Workers with DM	General review	Blanket employment policies that disqualify workers with DM are unnecessary in many occupational fields. In assessing occupational risks and fitness for duty in workers with DM, it is important to perform an individualized assessment of the worker and consider the risk factors for hypoglycemia, information from the treating clinician, essential job functions, and availability of reasonable accommodations (if needed).
(22) Jenni Ervasti, Marianna Virtanen, Jaana Pentti, Tea Lallukka, Petter Tinghög, Linnea Kjeldgard, Ellenor Mittendorfer-Rutz, and Kristina Alexanderson, published in 2015 [38]	Nationwide Swedish registers and linked, Sweden	Cohort study	Individuals with incident DM and individuals without DM	This Swedish population-based cohort study with registered data included 14,428 individuals with incident DM in 2006 and 39,702 individuals without DM from 2003 to 2009.	Work disability was substantially higher among people with DM (overall mean = 95 days per year over 7 years, SD = 143) than among those without DM (mean = 35 days, SD = 95). The risk of work disability was slightly higher after DM diagnosis than before and compared with the risk of those without DM. The trajectory of work disability was already increasing before diagnosis, increased more at the time of diagnosis, and plateaued after diagnosis. Individual sociodemographic characteristics and comorbidities were taken into account
(23) Kimberly J. Smit, Sonya S. Deschênes, and Norbert Schmitz, published in 2018 [35]	Diabetes Center, UK	Systematic review	All studies about diabtets	We searched seven databases for studies examining the longitudinal relationship between anxiety and DM. Two independent reviewers screened studies from a population aged 16 or older that examined either anxiety as a risk factor for incident DM or DM as a risk factor for incident anxiety. In addition, studies that met eligibility criteria were put forward for data extraction and meta-analysis.	There was an association between baseline anxiety and incident DM. The results also indicated the need for more research to examine the direction of association from DM to incident anxiety. This work adds to the growing body of evidence that poor mental health increases the risk of developing DM.
(24) Nao Sonoda, Soichiro Watanabe, Yuko Ohno, Kayo Godai, Chieko Hatamochi, Yoshie Sugimoto, Satoko Okawa, Maiko Shikama, and Akiko Morimoto, published in 2019 [28]	Institutional Review Board of Osaka University, Japan	Self-administered questionnaire and specific health checkup data	Employees with DM	This cross-sectional study was conducted in 2018. Participants were 140 full-time employees with T2DM aged over 40 years. Participants were classified into two groups: a dropout group and a continuation group. Work-related, personal, and DM-related factors were evaluated using a self-administered questionnaire and specific health checkup data.	Our findings suggest supervisor support, age, and metabolic syndrome are important factors related to dropout from outpatient DM treatment visits among Japanese male employees with DM.
(25) Isabela Fernandes de Aguiar Tonetto, Marcelo Henrique Barbosa Baptista, Danielle dos Santos, Gomides and Ana Emilia Pace, published in 2019 [30]	Study carried out in primary, secondary, and tertiary health care units with individuals in outpatient care, Brazil	The validated Diabetes-39 instrument	A total of 53 people with T2DM	This was a quantitative, cross-sectional, and descriptive study. The sample consisted of 53 people with T2DM.	QoL tends to worsen as the disease worsens. The results suggest QoL is related to sociodemographic and clinical variables. Therefore, these should be considered in the provision of care.
(26) Rose Nabi Deborah Karimi Muthuri, Flavia Senkubuge, and Charles Hongoro, published in 2020 [16]	Public and mission hospitals in the Meru County of Kenya	The EuroQol five-dimension five-level instrument (EQ-5D-5L)	A total of 32 HCWs	A cross-sectional study design was undertaken among 553 HCWs across 24 hospitals in Meru County.	Personal, job-related attributes and work environment characteristics are significant predictors of HCWs’ HRQoL. Thus, health policymakers and managers must consider these factors when developing and implementing policies and programs aimed at promoting HRQoL among HCWs.
(27) Evangelia Nena, Maria Katsaouni, Paschalis Steiropoulos, Evangelos Theodorou, Theodoros C Constantinidis, and Grigorios Tripsianis, published in 2018 [15]	Tertiary university hospital in Greece	WHO-5 Well-Being Index (WHO-5), a questionnaire on demographics and medical history, and the Shift Work Disorders Screening Questionnaire (SWDSQ)	HCWs	This was a cross-sectional study of HCWs: 312 employees working either in an irregular shift system or exclusively in morning shifts. All participants answered thequestionnair Shift workers completed the SWDSQ.	Most shift workers (58.2%) were somehow or totally dissatisfied with their sleep quality. Regression analysis revealed the following independent determinants for sleep impairment: parenthood, age 36–45 years, night shifts/week, and working more than 5 years in an irregular shift system. In addition, DM was the most common medical condition reported by shift workers. Comparison between the two groups revealed significant impairment in the WHO-5 total score and 4 of 5 items. Thus, shift work impairs QoL, whereas employees’ duration, frequency, age, and family status can adversely affect sleep.

**Table A2:** Quality Assessment of Included Studies.

Authors’ Name(s)	Title and Abstract	Introduction and Aim	Methodology and Sampling Data	Analyzing the Data	Bias and Ethics	Results	Transferability/Generalizability	Implications	Usefulness	Total Score /36
Coetzee et al., 2019 [17]	4	4	3	3	3	4	3	3	3	30
Hegde et al., 2015 [18]	4	4	4	4	3	4	2	3	3	31
Huang et al., 2016 [19]	4	4	4	4	3	4	3	3	3	32
Hansen et al., 2018 [6]	4	4	3	3	3	4	3	3	3	30
Manodpitipong et al., 2017 [21]	3	4	4	4	3	4	3	3	3	31
Nakao et al., 2021 [22]	4	4	4	4	3	4	3	3	3	32
Seuring et al., 2015 [24]	4	4	3	4	2	4	2	2	3	32
Hudson et al., 2016 [14]	4	4	4	4	4	4	4	3	4	35
Olesen et al., 2020 [23]	4	4	2	2	1	3	2	3	2	24
Hakkarainen et al., 2016 [26]	4	4	4	4	3	3	2	4	3	34
Loerbroks et al., 2018 [27]	4	4	4	4	3	4	3	4	4	34
Binesh et al., 2021 [31]	4	4	4	4	3	4	2	3	3	31
Abu et al., 2016 [32]	4	4	4	4	4	4	2	3	3	32
Galarraga & Llahana, 2018 [33]	4	4	4	4	3	4	2	4	4	33
Pasmooij et al., 2016 [25]	4	3	4	4	4	4	4	3	3	33
Smith et al., 2018 [35]	4	4	4	4	3	4	2	2	3	30
Nexø et al., 2020 [37]	4	4	3	4	3	4	2	3	3	34
Gerbo et al., 2019 [34]	4	4	3	4	2	4	2	3	3	29
Hansen et al., 2016 [20]	4	4	4	4	3	4	3	3	3	32
McCarthy et al., 2021 [40]	4	4	4	4	4	3	4	4	4	35
Ervasti et al., 2015 [38]	4	4	4	4	3	4	3	3	3	32
Nielsen et al., 2016 [29]	4	4	4	4	2	4	3	3	4	32
Imbroll & Cassar, 2021 [36]	4	4	3	4	3	4	3	4	4	33
Ervasti et al., 2016 [39]	4	4	4	3	3	4	4	3	3	32
Tonetto et al., 2019 [30]	4	4	4	4	3	3	4	3	3	32
Nena et al., 2018 [15]	4	4	4	4	4	3	3	3	3	32
Muthuri et al., 2021 [16]	4	4	4	4	4	3	4	3	4	34
